# Machine-learning and combined analysis of single-cell and bulk-RNA sequencing identified a DC gene signature to predict prognosis and immunotherapy response for patients with lung adenocarcinoma

**DOI:** 10.1007/s00432-023-05151-w

**Published:** 2023-07-28

**Authors:** Liangyu Zhang, Maohao Guan, Xun Zhang, Fengqiang Yu, Fancai Lai

**Affiliations:** 1 Department of Thoracic Surgery, The First Affiliated Hospital, Fujian Medical University, Fuzhou, 350005 China; 2 Department of Thoracic Surgery, National Regional Medical Center, Binhai Campus of the First Affiliated Hospital, Fujian Medical University, Fuzhou, 350212 China

**Keywords:** Single-cell, Dendritic cell (DC), Machine-learning, Prognosis, Immunotherapy

## Abstract

**Background:**

Innate immune effectors, dendritic cells (DCs), influence cancer prognosis and immunotherapy significantly. As such, dendritic cells are important in killing tumors and influencing tumor microenvironment, whereas their roles in lung adenocarcinoma (LUAD) are largely unknown.

**Methods:**

In this study, 1658 LUAD patients from different cohorts were included. In addition, 724 cancer patients who received immunotherapy were also included. To identify DC marker genes in LUAD, we used single-cell RNAsequencing data for analysis and determined 83 genes as DC marker genes. Following that, integrative machine learning procedure was developed to construct a signature for DC marker genes.

**Results:**

Using TCGA bulk-RNA sequencing data as the training set, we developed a signature consisting of seven genes and classified patients by their risk status. Another six independent cohorts demonstrated the signature’ s prognostic power, and multivariate analysis demonstrated it was an independent prognostic factor. LUAD patients in the high-risk group displayed more advanced features, discriminatory immune-cell infiltrations and immunosuppressive states. Cell–cell communication analysis indicates that tumor cells with lower risk scores communicate more actively with the tumor microenvironment. Eight independent immunotherapy cohorts revealed that patients with low-risk had better immunotherapy responses. Drug sensitivity analysis indicated that targeted therapy agents exhibited greater sensitivity to low-risk patients, while chemotherapy agents displayed greater sensitivity to high-risk patients. In vitro experiments confirmed that CTSH is a novel protective factor for LUAD.

**Conclusions:**

An unique signature based on DC marker genes that is highly predictive of LUAD patients’ prognosis and response to immunotherapy. CTSH is a new biomarker for LUAD.

**Supplementary Information:**

The online version contains supplementary material available at 10.1007/s00432-023-05151-w.

## Introduction

Globally, lung cancer ranks first in cancer-related deaths (Sung et al. 2021), with lung adenocarcinoma (LUAD) representing the major histological subtype (Little et al. 2007; Chang et al. 2015). There has been considerable progress in therapeutic approaches for LUAD, but the 5-year overall survival is still below 20% (Brahmer et al. 2012).

Treatment paradigms for LUAD have shifted dramatically over the years due to the increase in clinical effectiveness of immunotherapies, a treatment that encourages immune cells in the tumor microenvironment to kill malignant cells by blocking the expression of immune checkpoints (Imielinski et al. 2012; Topalian et al. 2012). The heterogeneity of tumor microenvironment (TME), however, is not fully reflected by some of the biomarkers used to predict immunotherapy responses, such as PD-L1, and patients with NSCLC who receive immunotherapy have a 20% response rate (Gibney et al. 2016; Sharma et al. 2017). This makes identifying new biological markers and developing prediction models essential for predicting prognosis and therapeutic effect.

During cancer progression, TME plays a significant role, and immunotherapy can be heavily influenced by TME (Hinshaw and Shevde 2019; Xiao and Yu 2021). Anti-tumor immunity focuses primarily on the adaptive T cell responses, while innate immunocytes, such as NK cells, phagocytes, and dendritic cells, are underrepresented. There is increasing recognition of dendritic cells (DCs), which are important antigen processing cells originated from bone marrow and disseminated throughout the body, as key players in immune response (Kastenmüller et al. 2014). APCs such as macrophages are less effective than DCs at eliciting response from T cells (Steinman 2012; Steinman and Cohn 1973), which is why DCs have earned the nickname ‘professional APCs’. Apoptotic and necrotic tumor fragments can be taken up and processed by DCs, and tumor-antigens can be presented to T cells by DCs (Fuertes et al. 2011; Palucka and Banchereau 2012). In addition, DCs can sample tumor antigens by nibbling live tumor cells (Dhodapkar et al. 2008). Hence, DC modulation is highly sought after for improving cancer immunotherapy, and in the development of cancer vaccines, DCs are considered to be the most important target cells (Wculek et al. 2020; Ueno et al. 2010). DCs have been studied extensively in the field of health and disease (Morante-Palacios et al. 2021; Worbs et al. 2017; Wilensky et al. 2014; Dou et al. 2018; Parenti et al. 2017), but there is little information on the molecular analysis of DCs in LUAD.

With the advent of single-cell RNA sequencing and its associated data analysis methods, it is now possible to decipher the molecular characteristics of different immunity cells in a way that has never before been possible (Chen et al. 2019). According to previous researches, using scRNA-seq data to identify gene expression profiles in immunocytes may be a powerful method of predicting prognoses and immunotherapy efficacy for cancer patients (Liang et al. 2021; Song et al. 2022a, b). As a first step, we analyzed single-cell RNA sequencing data for LUAD to identify DC markers. Dendritic Cell Implicated Risk Scores (DCIRS) were derived from bulk RNA-seq analyses of samples with LUAD to predict prognoses. Moreover, DCIRS was validated in six independent cohorts from GEO, which demonstrates its predictive power. In addition, DCIRS was investigated in relation to LUAD’s immunotherapy, targeted therapy, and chemotherapy response. Finally, we identified a new gene, CTSH, is a protective factor for LUAD. It might exerts its protective effect by inhibiting tumor cells’ migration, invasion, proliferation, epithelial-mesenchymal transition, and promoting tumor cells’ apoptosis.

## Materials and methods

### Data acquired

Single-cell RNA-sequencing data of five primary LUAD samples from the EMTAB6149 dataset was downloaded from the TISCH (Sun et al. 2021) database to determine the genes associated with dendritic cells (DCs). UCSC Xena was used to download TCGA (Hugo et al. 2016) bulk tumor transcriptomic data for 497 patients with LUAD and their corresponding clinical information. And for external validation, six microarray datasets, namely GSE11969, GSE41271, GSE50081, GSE42127, GSE72094 and GSE31210, were obtained from the GEO database (Clough and Barrett 2016). Table S1 shows the clinical characteristics of these 6 GEO cohorts. GISTIC2.0 (genepattern) (Mermel et al. 2011) was implemented to analyze Copy number Cariation (CNV) data, and the analysis of somatic mutation data from TCGA was performed by the Sangerbox website (Shen et al. 2022). Multi-omics data, such as neoantigen and aneuploidy data, proliferation and wound healing information, were acquired from Thorsson V’s study (Liu and Wu 2018). From the GEO database, the dataset GSE114761 containing EMT data for A549 LUAD cell line was downloaded. Survival meta-analysis was conducted using LCE database.

TCGA-LUAD (*n *= 497) and GSE72094 (*n* = 397) were used for interactive verification in the subsequent analysis of immunoinfiltration, immunotherapy, and drug sensitivity, due to their largest sample sizes.

### Identifying DC marker genes by scRNA-seq

ScRNA-seq data were dealt with the “Seurat” R package. The raw matrix of scRNA-seq data was filtered using three methods to retain high-quality data: cells with fewer than 200 genes or more than 2500 genes or more than 5% mitochondrial genes were excluded, and genes expressed in at least three cells were included. Using Seurat’s “NormalizeData” function, we first normalized scRNA-seq data by setting the normalization method to “LogNormalize”. A Seurat object was created from normalized scRNA-seq data, and the “FindVariableFeatures” function was used to identify the top 2000 highly variable genes. In the subsequent steps, we applied the “RunPCA” function to reduce the dimension of the scRNA-seq data by performing a principal component analysis. “FindNeighbors” and “FindClusters” functions were used for cell clustering. Next, UMAP was performed and UMAP_1 and UAMP_2 were used to demonstrate cell clustering. As a means of annotation, we used the R package “SingleR” (Aran et al. 2019) along with annotated cell data from the TISCH database. The “FindAllMarkers” function in Seurat package was then used to identify genes that were differentially expressed in DCs compared to other cells, and adjusted *p* value < 0.01 and |log2 (fold change)|> 1 were considered as DC marker genes.

### Machine learning algorithms

Using ten machine learning algorithms, we developed a consensus dendritic cell implicated risk score (DCIRS). A number of algorithms were incorporated into the integrated model, including RSF, Enet, Lasso, stepwise Cox, Ridge, CoxBoost, plsRcox, SuperPC, GBM, and survival SVM. For selecting the model with the highest average C-index across all validation sets, we also combined these algorithms, including 90 different combinations. To test DCIRS’ prognostic value, Boruta, RSF, and GBM were applied. Table 1 describes the R packages by which each algorithm is implemented.

**Table 1:** Description of R packages that implement machine learning algorithms

Algorithms	R packages
(Random survival forest) RSF	RandomForestSRC
(Generalized boosted regression modeling) GBM	gbm
(Least absolute shrinkage and selection operator) Lasso	glmnet
Ridge	glmnet
(Elastic network) Enet	glmnet
CoxBoost	CoxBoost
Stepcox	survival
(Supervised principal components) SuperPC	superpc
(Survival support vector machine) survival-SVM	survivalsvm
(Partial least squares regression for Cox) plsRcox	plsRcox
Boruta	Boruta

To validate DCIRS’s predictive value, the area under the curve (AUC) was calculated using the “timeROC” package. Cox regression analysis was carried out using the R package “survival” to validate DCIRS’s independent prognostic significance.

### Immune infiltration analysis and gene enrichment analysis

To determine the relative number of immune cells infiltrating the TCGA-LUAD and GSE72094 cohorts, six algorithms were used: ssGSEA, TIMER, quanTIseq, MCP-counter, EPIC, and ESTIMATE. R package "GSVA" was used to implement ssGSEA algorithm (Hänzelmann et al. 2013), and R package “IOBR” was used for implementing the other five algorithms (Zeng et al. 2021). Additionally, we calculated DCIRS correlations with various immune pathways using ssGSEA. DESeq2 was used to identify differentially expressed genes (DEGs) in patients between different DCIRS groups, and defining DEGs was based on* P*adj < 0.05 and |log2(fold-change)|> 1. Furthermore, the R package “clusterProfiler” was used to conduct GO_KEGG and GSEA enrichment analysis (Yu et al. 2012). “h.all.v7.4.symbols” and “c2.cp.kegg.v7.4.symbols” are selected as two reference gene sets. R package “ComplexHeatmap” was used to generate each heatmap plot.

In previous work, six immune subsets (C1–C6) were proposed. This work replicates that classification by using the R package ‘ImmuneSubtypeClassifier’.

### Cell–cell communication analysis

Analysis of cross-chat between immune cells and LUAD cells is conducted by the R package ‘CellChat’ (Jin et al. 2021), which identifies differential ligand-receptor pairs.

### Immunotherapy efficacy prediction

First, we used PD-L1 expression, TCR repertoires, TIDE (Jiang et al. 2018) and IPS algorithms to predict immune checkpoint blockade response. The TCGA-LUAD cohort’s RNA-sequencing data were used to determine PD-L1 mRNA expression levels. Diversification of the repertoire of TCRs was measured by richness and Shannon diversity indices. The TCR is responsible for recognizing antigens presented by the MHC, making TCR repertoire analysis an important biomarker for stratification and monitoring ICB therapy. In Thorsson V’s study, the abundance of TCR were obtained for LUAD patients in TCGA cohort. Using the TIDE website, we analyzed LUAD patients’ tumor immune dysfunction and exclusion (TIDE) scores. A higher TIDE score indicates a less effective ICB therapy. LUAD patients’ Immunophenotypescore (IPS) were downloaded from the TCIA database (Hugo et al. 2016), and ICB therapy response was positively related to IPS. Further validation was conducted in the actual immunotherapy cohort. Two immunotherapy cohorts, including GSE126044 (NSCLC) and GSE35640 (melanoma), were acquired from the GEO database. Besides, from the TIGER database (Chen et al. 2022), four immunotherapy cohorts were acquired, including GSE91061 (melanoma), PRJE23709 (melanoma), phs000452 (melanoma) and PRJNA482620 (GBM). From these ICB-treated cohorts, we retrieved clinical information and RNA sequencing data to speculate DCIRS’s potential value for predicting immunotherapy response. 121 ICB-treated melanoma patients’ expression profile and clinical information were retrieved from the study of Liu et al. (PMID: 31792460, Dec 25, 2019); and 152 ICB-treated NSCLC patients’ expression profile and clinical information were retrieved from the study of Ravi A et al. (PMID: 37024582, Apr 6, 2023).

In addition, two sc-RNA sequencing sets containing immunotherapy data, namely GSE123813 (basal cell carcinoma, BCC) and GSE145281 (Bladder Carcinoma, BLCA), were downloaded from the TISCH database and processed by the Seurat package as above described.

### Prediction of potential sensitive drugs

Information about drug sensitive data, as well as corresponding gene expression matrix, were obtained from the Cancer Therapeutics Response Portal version 2 (CTRP2). IC50, which is a measure of a cells’ sensitivity to drugs, is often used to determine a potential drug’s sensitivity. A lower IC50 suggests a higher sensitivity. In this study, the IC50 value for each sample was calculated using the R package ‘oncoPredict’ (Maeser et al. 2021). Data on the sensitivity of NSCLC cell lines to gefitinib and erlotinib were acquired from the GSE34228 and GSE31625 datasets in GEO.

### Cell culture and transfection

Normal bronchial epithelial cell line BEAS-2B and three LUAD cell lines A549, HCC827, and H1975 was purchased from the Cell Bank of the Chinese Academy of Sciences. At the condition of 37 °C and 5% CO_2_, all cells were cultured in RPMI-1640 medium supplemented with 10% fetal bovine serum (FBS) (Gibco, USA). Small interfering RNA (si-RNA), including si-CTSH and its negative control, si-NC, were purchased from Hanheng Biology (Shanghai, China). Lipofectamine 3000 (Invitrogen, Carlsbad, CA, USA) was used to transfect the siRNA into cells as described by the manufacturer.

### RNA extraction and RT-PCR

Total RNA was extracted using RNA extraction kit (Vazyme, China) according to the manufacturer’s instructions. An All-in-One First-Strand Synthesis MasterMix kit (iScience, China) was then used to reverse transcribe the total RNA sample into cDNA. Triplicate aliquots of each cDNA sample were subjected to RT-qPCR performed using Taq SYBR^®^ Green qPCR Premix (iScience, China). The internal reference was GAPDH.

The primers used for CTSH was as follows: forward:5′-TGCCTTTGAGGTGACTCAGG-3′; reverse: 5′-GCGCTCGATGAGGAAGTACC-3′. The primers used for GAPDH was as follows: 5′-GGTGTGAACCATGAGAAGTATGA-3′, reverse: 5′-GAGTCCTTCCACGATACCAAAG-3′.

### Western blotting

Using a RIPA lysis buffer (Meilun Biotechnology, China), total protein was extracted from cells. A bicinchoninic acid protein assay kit (#23227; Thermo Fisher Scientific, Waltham, USA) was used to measure protein concentration. Denatured proteins were subject to 10% SDS-PAGE and blotted onto nitrocellulose membranes (Millipore, Bedford, USA). After being blocked with 5% skimmed dry milk for 2 h, the membranes were subsequently incubated with primary antibodies, including anti-CTSH (1:1000, Immunoway), and anti-β-Actin (1:1000, Immunoway) overnight at 4 °C, then incubation with horseradish peroxidase labeled secondary antibodies (ab7090, 1:5000; Abcam) followed. β-Actin was used to normalize the expression of target proteins.

### Transwell assays

Using Transwell chambers (Scipu002872; Corning Inc., Corning, USA) with inserts coated with Matrigel, invasion assays were conducted. In the upper chamber, 50,000 cells with 10% FBS were seeded, and 600 μl of 20% FBS were added in the lower chamber. Cotton swabs were used to clean the upper membrane surface after incubation for 24 h. Polyformaldehyde was used to fix the cells passing through the membrane, and then hematoxylin was used to stain them. Counting invaded cells was done using a high-powered microscope.

### Wound healing assays

The HCC827 cells were seeded at a density of 10^5^ cells per well in six well plates. A 10-μl pipet tip was used to scratch the cell monolayers after 24 h of incubation when the cells were approximately 80% confluent. Using PBS, detached cells were washed from scratched monolayers. For reference, the dish’s bottom was marked. Each sample’s wound area was recorded at 0 h and 36 h, and ImageJ software was used to analyze the images quantitatively. The formula: {(initial area − final area)/initial area} × 100%, was used to calculate wound closure rate.

### Cell apoptosis analysis

Apoptosis was assessed using an Annexin V-APC/7-AAD Apoptosis Detection Kit (KeyGEN, China). As a result of the treatment, the cells were harvested, washed twice with ice-cold PBS, and added 500 μl of binding buffer to create a single cell suspension. Afterward, Annexin V-APC and 7-AAD were added in darkness for 15 min. Apoptosis was detected by flow cytometry. Apoptosis is defined as cells staining positive for Annexin but negative for 7-AAD or staining positive for both.

### Statistical analysis

All experiments were performed at least three times. For comparison between two groups, the Wilcoxon test or *t* test were used. Spearman correlation was used for correlation analyses. K–M analysis was used to predict the overall survival difference between the low and high DCIRS groups. The prognostic value of DCIRS and clinicopathological characteristics was investigated using multivariate and univariate Cox regression analyses. With the “timeROC” R package, receiver operating characteristic curves (ROC) are analyzed in time-dependent manner. The Hmisc R package was used to compute the C-index, and the compare C R package was used to compare C-indexes between groups. Ns—*P* ≥ 0.05, *P < 0.05, **P < 0.01, ***P < 0.001. All statistical analysis was conducted in R (v 4.1.1).

### Databases’ websites

TCGA GDC: http://cancergenome.nih.gov/.

UCSC Xena: https://xena.ucsc.edu/.

GEO: https://www.ncbi.nlm.nih.gov/geo/.

TISCH: http://tisch.comp-genomics.org.

TIGER: http://tiger.canceromics.org/#/.

TCIA: https://tcia.at/home.

TIDE: http://tide.dfci.harvard.edu.

Sangerbox: http://sangerbox.com.

CTRP2: https://portals.broadinstitute.org/ctrp.

LCE: https://lce.biohpc.swmed.edu/lungcancer/index.php#about.

## Results

### Identification of DC marker genes

**Fig. 1:**
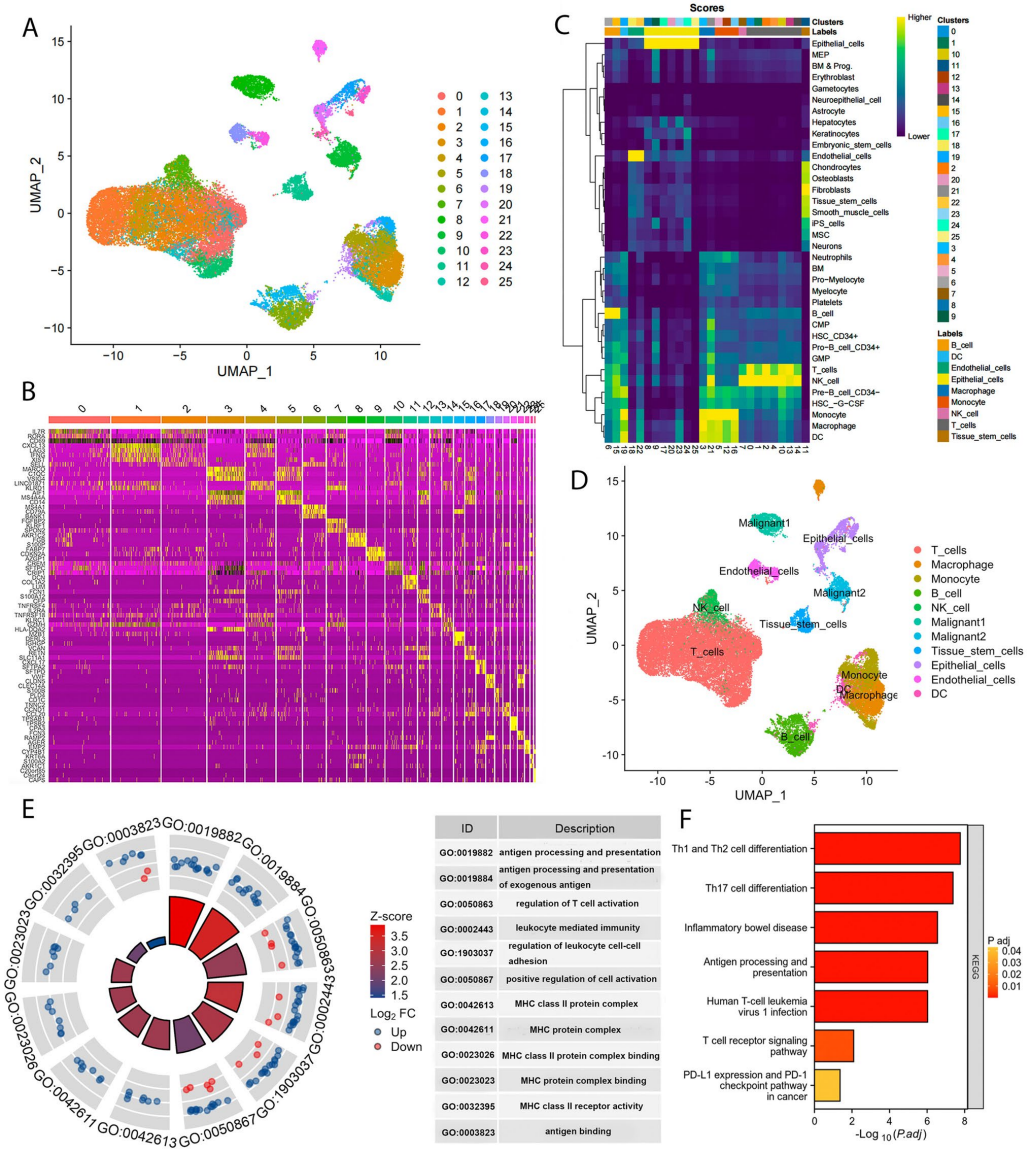
Identification of DC marker genes using sc-RNA sequencing. **A** UMAP plot colored by 25 clusters of cells. **B** An overview of each cluster’s top 3 marker genes. **C** SingleR-annotated heat map. **D** Annotated 12 cell types. Gene enrichment analysis of DC marker genes using GO (**E**) and KEGG (**F**)

As a result of data processing and screening, gene expression profiles from 40,218 cells were obtained from five LUAD samples. Based on the top 2000 variable genes, we conducted PCA to reduce the dimensionality, resulting in the identification of 26 cell clusters (Fig. 1A). Heatmaps display the relative expression of marker genes within clusters (Fig. 1B). In addition, cells in cluster 19 were considered DCs by annotating them using the Human Primary Cell Atlas reference dataset (Fig. 1C, D). As shown in Table S2, 83 DC marker genes were identified. Gene enrichment analysis indicated that DC marker genes were mainly enriched in some immune related functions, such as antigen processing and presentation, MHC protein complex binding, leukocyte mediated immunity, and PD-L1 checkpoint pathway in cancer (Fig. 1E, F).

### Construction of dendritic cell implicated risk score (DCIRS) by integrated machine learning

**Fig. 2:**
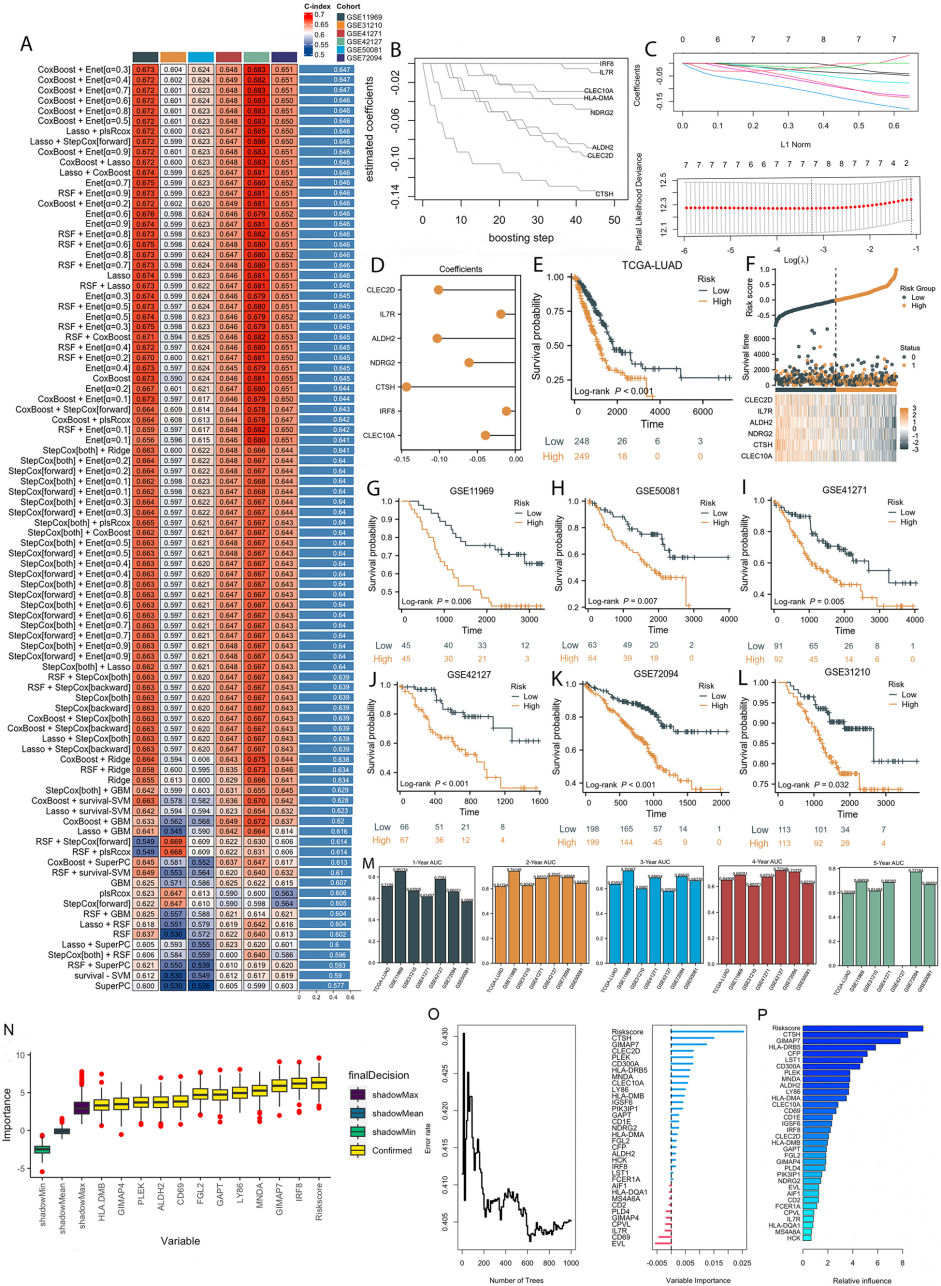
Developing a gene signature by machine learning based on DC marker genes and testing its robustness. **A** A total of 91 different models were built by machine learning and their C-indexes were tested in each verification set. **B** Eight key genes identified by CoxBoost. **C** Seven key genes identified by Enet. **D** Seven genes’ coefficients calculated by Enet. **E** Patients’ different prognosis among different risk groups at TCGA set. **F** Distribution of risk scores and survival status, as well as 7 DC marker genes’ expression characteristics. Patients’ different prognosis among different risk groups at GSE11969 (**G**), GSE50081 (**H**), 41271 (**I**), GSE42127 (**J**), GSE72094 (**K**), and GSE31210 (**L**) sets. **M** An assessment of the DCIRS’s ability to predict patients’ survival at 1, 2, 3, 4, and 5 years. The importance of DCIRS and DC marker genes for the prognosis of LUAD patients by Boruta (**N**), RSF (**O**), and GBM (**P**)

Based on the univariate Cox analysis, 33 of the 83 DC markers were found to be prognostic genes (Table S3). Our machine learning-based procedure was applied to these 33 genes to develop a signature. TCGA dataset was fitted with 90 prediction models, and the C-index was calculated across six validation datasets. Finally, the combination of CoxBoost and Enet (*α* = 0.2) produced the optimal model with a average C-index of 0.647 across all validation sets (Fig. 2A). Using CoxBoost algorithm, 8 key genes were identified, and Enet algorithm selected seven of them to calculate the risk score (Fig. 2B, C). As shown in Fig. 2D, the Enet algorithm also obtained the coefficients of each of the 7 genes. Based on the expression of seven genes weighted by their regression coefficients, a dendritic cell implicated risk score (DCIRS) was calculated for each patient.

$$\begin{aligned} {\text{DCIRS }} &= {\text{ IL7R }} \times \, \left( { - 0.02084432} \right) \, + {\text{ IRF8 }} \times \, \left( { - 0.01001391} \right) \, \hfill \\ &\quad + {\text{ CLEC10A }} \times \, \left( { - 0.03966908} \right) \, + {\text{ CLEC2D }} \times \, \left( { - 0.10397921} \right) \hfill \\ \, &\quad+ {\text{ CTSH }} \times \, \left( { - 0.1457732} \right) \, + {\text{ ALDH2 }} \times \, \left( { - 0.10492581} \right) \hfill \\ \, &\quad+ {\text{ NDRG2 }} \times \, \left( { - 0.06235225} \right). \hfill \\ \end{aligned}$$

DCIRS increases with OS decreasing, while mortality increases (Fig. 2F). The DCIRS medium value was used to categorize patients into high- and low-risk groups. The TCGA training dataset and six validation datasets showed that the high-risk group had significantly poorer survival than the low-risk group (all *P* value less than 0.05, Fig. 2E, G–L). Multivariate cox analysis demonstrates that despite adjusting for clinical traits, DCIRS remained statistically significant in all cohorts (all *P* value less than 0.05), suggesting that DCIRS is an independent risk factor for OS (Tables S4–S10). Based on the ROC analysis, DCIRS discriminated well with one-, two-, three-, four-, and five-year AUCs of 0.711, 0.617, 0.635, 0.649, and 0.593 in the TCGA-LUAD; 0.851, 0.741, 0.744, 0.688, 0.690 in GSE11969; 0.670, 0.624, 0.599, 0.604, 0.615 in GSE31210; 0.615, 0.683, 0.687, 0.674, 0.684 in GSE41271; 0.778, 0.704, 0.579, 0.723, in GSE42127; 0.661, 0.689, 0.696, 0.713, 0.772 in GSE72094; 0.569, 0.643, 0.664, 0.622, 0.667 in GSE50081 (Fig. 2M). As indicated by these indicators, DCIRS performed well in multiple independent cohorts.

We also used three machine-learning algorithms, including Boruta (Fig. 2N), RSF (Fig. 2O), and GBM (Fig. 2P), to compare the importance of DCIRS and DC marker genes for prognosis in LUAD patients. According to the results, DCIRS had the greatest effect on LUAD patients’ prognosis.

### Nomogram

**Fig. 3:**
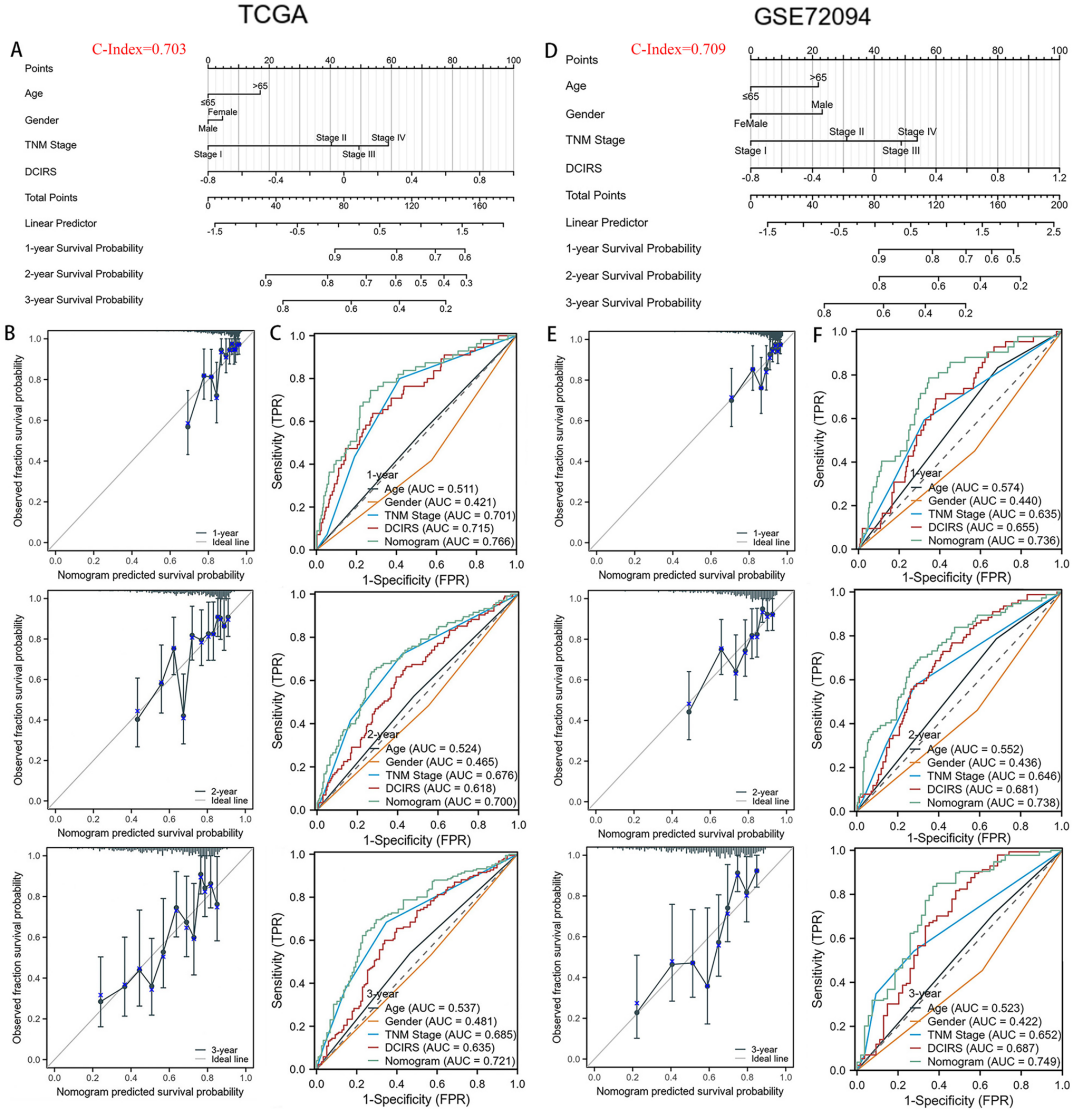
Construction of the nomogram. Construction of the nomograms from TCGA (**A**) and GSE72094 (**D**) datasets. Calibration curves for nomograms constructed from TCGA (**B**) and GSE72094 (**E**). ROC curves for nomograms constructed from TCGA (**C**) and GSE72094 (**F**)

Nomogram was constructed by combining clinical parameters with DCIRS from the TCGA dataset (Fig. 3A), and GSE72094 dataset was selected for validation based on its largest sample size across all validation sets (Fig. 3D). It indicates that the incorporation of clinical characteristics improves the ability of DCIRS to predict patients’ survival, as illustrated by the ROC curves (Fig. 3C, F), and the calibration curves confirmed this nomogram’s accuracy (Fig. 3B, E).

### Differential analysis and function enrichment analysis

**Fig. 4:**
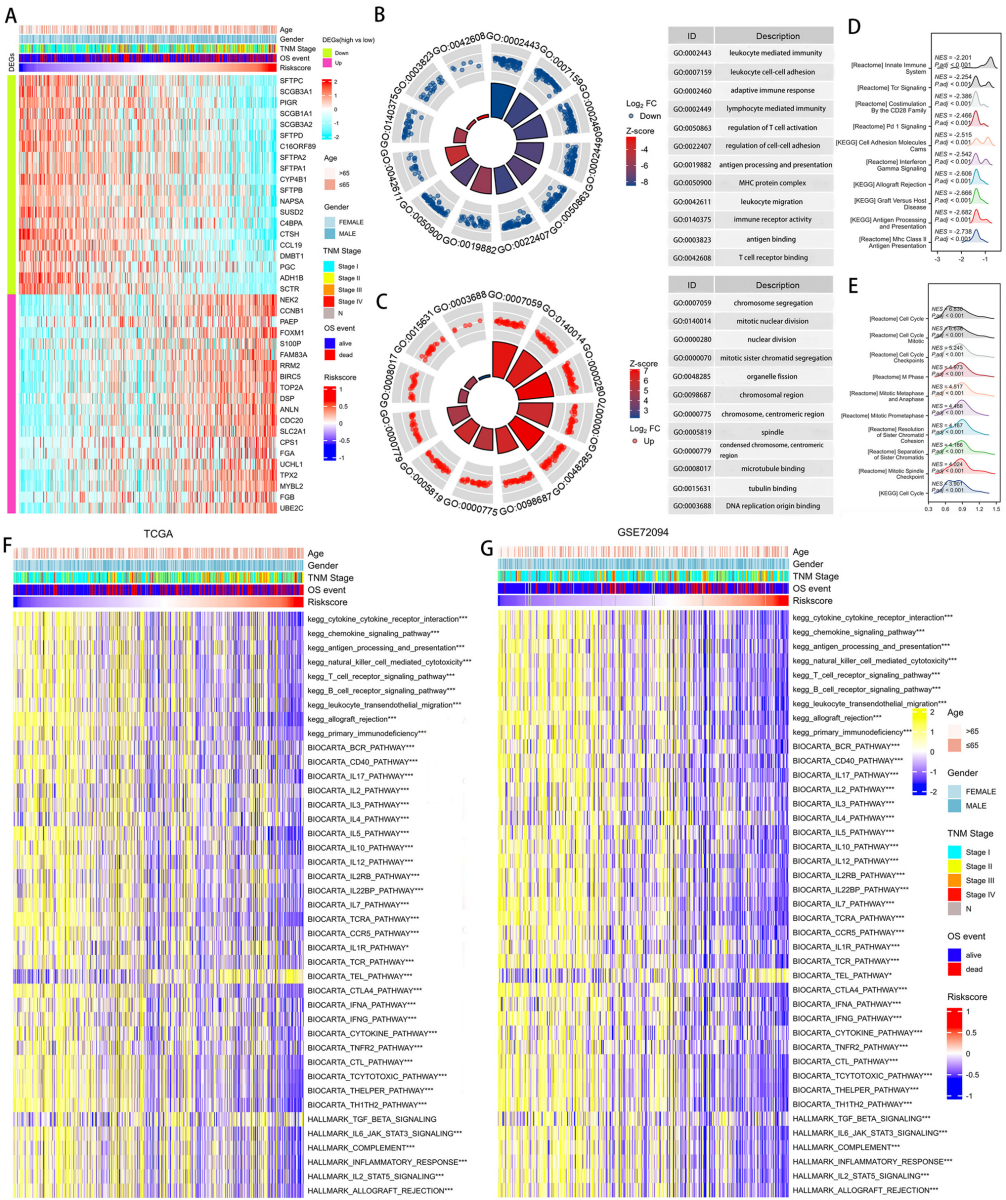
Investigation of DCIRS-related biological functions. **A** Expression profiles of the top 40 genes most positively or negatively correlated with DCIRS. GO analysis of genes with down-regulated (**B**) or up-regulated (**C**) expression in the high-DCIRS group compared with the low-DCIRS group. GSEA analysis of genes with down-regulated (**D**) or up-regulated (**E**) expression in the high-DCIRS group compared with the low-DCIRS group. Comparison of immune pathway activity between high and low DCIRS groups by GSVA in the TCGA (**F**) and GSE72094 (**G**) cohorts

Further studies were conducted to clarify the potential mechanism of DCIRS’ excellent predictive capability. Based on the results of differential analysis, we identified DEGs between groups with high and low DCIRS levels, and visualized the top 40 genes that differed most in terms of expression levels (Fig. 4A). TOP2A and FAM83A are known to play a carcinogenesis role in lung adenocarcinoma. We found that among high-risk patients, TOP2A and FAM83A are highly expressed. However, in low-risk individuals, protective factors like SFTPC and SUSD2 are highly expressed.

GO and GSEA analyses revealed that the up-regulated genes in the high-risk group were primarily involved in the biological functions such as chromosome segregation, nuclear division, microtubule binding, DNA replication origin binding, cell cycle, M phase, and mitotic spindle checkpoint, which are closely correlated with carcinogenesis (Fig. 4C, E). The down-regulated genes in the high-risk group were mainly involved in the functions such as leukocyte mediated immunity, adaptive immune response, antigen binding, regulation of T cell activation, TCR signaling, PD1 signaling, allograft rejection, and MHC II antigen presentation, which are immune-implicated functions (Fig. 4B, D). Low-risk patients also showed higher activity in immune-related pathways, such as TCR, BCR, IL2, IL3, IL4, IL5, IL17, CTLA4, CTL, and complement pathways, according to GSVA enrichment analysis in both TCGA and GSE72094 cohorts (Fig. 4F, G).

As a whole, high-risk patients exhibited more aggressive lung adenocarcinoma features, while low-risk patients exhibited immunoactive characteristics.

### Implications of DCIRS with tumor immune microenvironment (TIME)

**Fig. 5:**
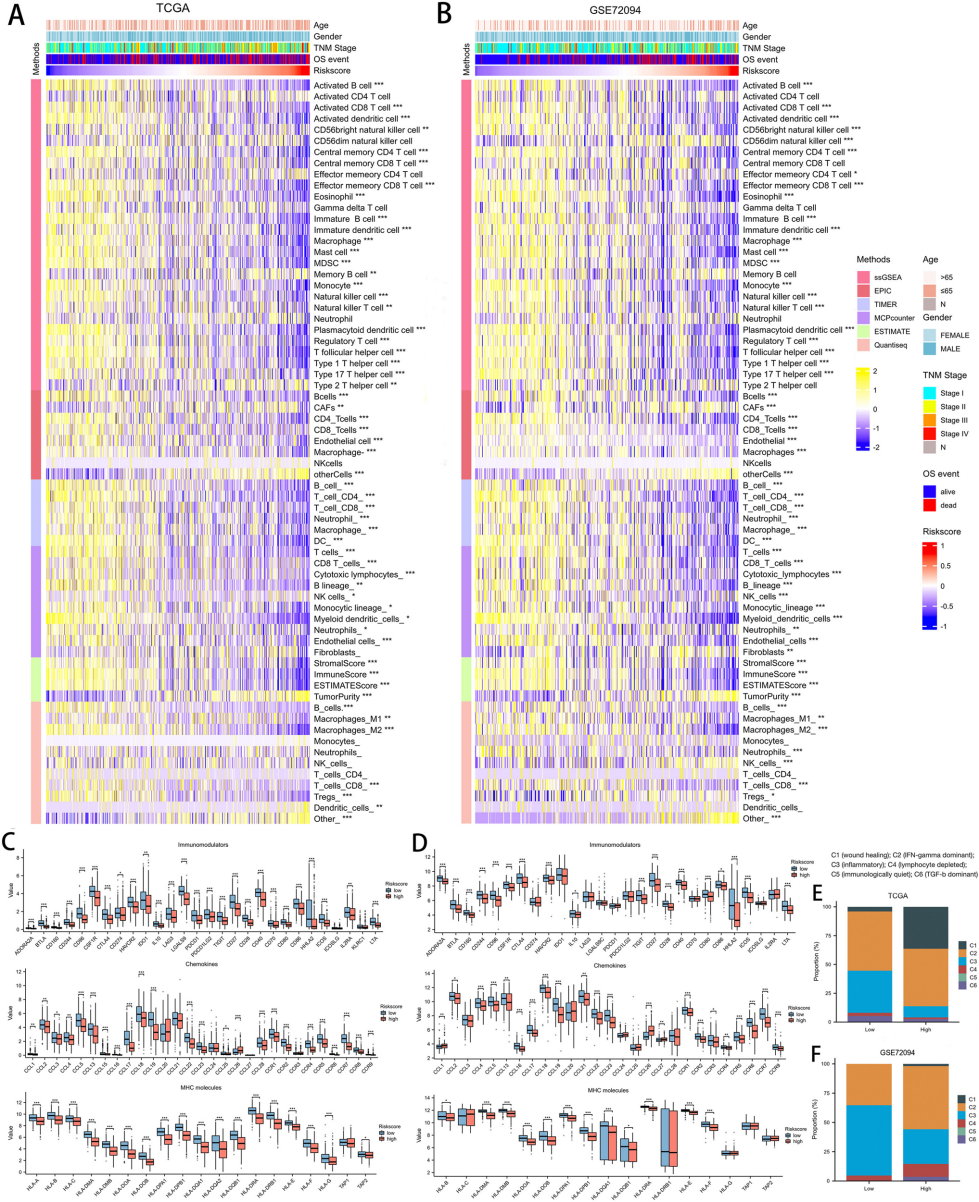
Comparison of immune characteristics between groups with high and low DCIRS. Quantification of different immune cell infiltration level between high and low DCIRS groups using six methods in the TCGA (**A**) and GSE72094 (**B**) sets. Immunogenes’ different expression between high and low DCIRS group in the TCGA (**C**) and GSE72094 (**D**) sets. Proportion of six immune subtypes between high and low DCIRS groups in the TCGA (**E**) and GSE72094 (**F**) sets

As low-risk patients exhibit immunoactive characteristics, we examined the relationship between DCIRS and TIME further. Based on immune cell infiltration analysis in TCGA and GSE72094, DCIRS and immune infiltration abundance were significantly inversely related, which means that patients in the low-DCIRS group had higher immunocyte infiltration level. The results were verified using six methods, including ssGSEA, TIMER, MCP-counter, ESTIMATE, EPIC, and quanTIseq (Fig. 5A, B). Besides, various immune genes were significantly higher expressed in low-DCIRS group, including immune checkpoints, such as PDCD1, CD274, CTLA4, TIGIT, and LAG3; chemokines, such as CCL1, CCL2, CCL3, CCL5, CCR1, and CCR2; MHC molecules, such as HLA-A, HLA-B, and HLA-C (Fig. 5C, D). Combined with the result of enrichment analysis, it suggests that patients with low-risk have a higher proportion of immune cells’ infiltration.

In addition, high-risk patients had a higher proportion of C1 subtype while had a lower proportion of C3 subtype than low-risk patients when analyzing the composition of immune subtypes (Fig. 5E, F). There was a high tumor cell proliferation rate and elevated expression of angiogenic genes in C1 (wound healing) subtype. In C3 (Inflammatory) subtype, tumor cell proliferation was low to moderate and aneuploidy levels were low. Patients with higher DCIRS tend to have a higher proportion of malignant phenotypes, which may partly explain their poorer prognosis.

### Ligand-receptor pairs between immunoocytes and LUAD cells

**Fig. 6:**
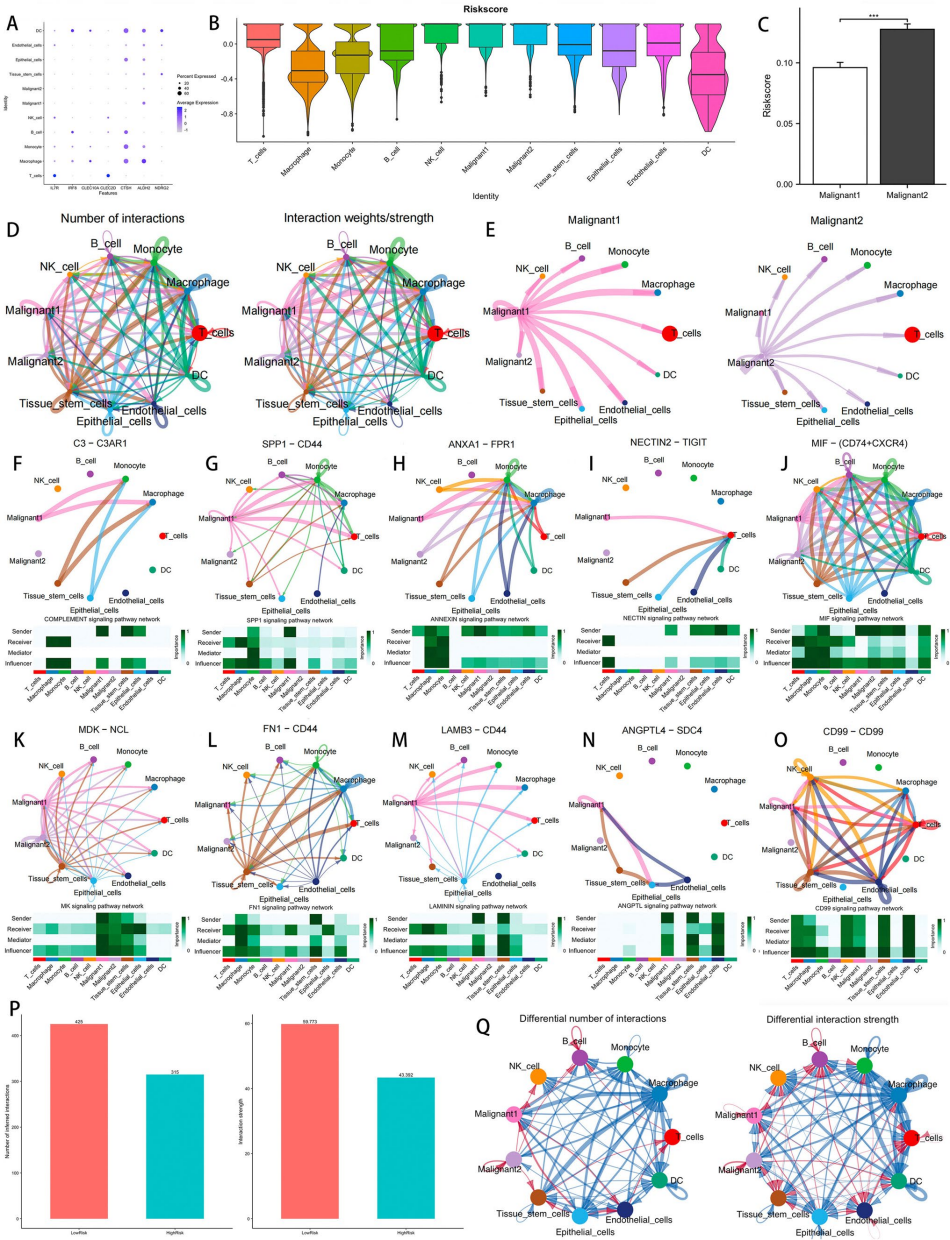
Analysis of cell-cell communication. **A** Expression of 7 genes that make up DCIRS at cellular level. **B** Distribution of DCIRS across all cell types. **C** Different DCIRS between malignant1 and malignant2. **D** Communication between all cells. **E** The ability of malignant1 and malignant2 to send signals to other cells. Communication between cells via the COMPLEMENT (**F**), SPP1 (**G**), ANNEXIN (**H**), NECTIN (**I**), MIF (**J**), MK (**K**), FN1 (**L**), LAMININ (**M**), ANGPTL (**N**), and CD99 (**O**) signaling pathway. **P** Comparison of the number and intensity of interaction between high and low DCIRS groups’ cells using bar plot. **Q** Comparison of the number and intensity of interaction between high and low DCIRS groups’ cells. The blue lines mean that the number/intensity of cell–cell communication was reduced in the high DCIRS group compared to the low DCIRS group, and the red lines mean that the number/intensity of cell–cell communication was increased in the high DCIRS group compared to the low DCIRS group

Patients with low DCIRS had higher immune cell infiltration and immune gene expression than those with high DCIRS. In order to investigate the potential mechanism behind this, we examined the communication between immunocytes and LUAD cells using scRNA-seq. First, we explored the expression of seven genes used to construct DCIRS at the cellular level (Fig. 6A), and calculated the risk scores of various cells, and found that malignant cells and NK cells were at the highest risk (Fig. 6B), and we found that compared to malignant 2, malignant 1 had a significantly lower risk score (Fig. 6C). Figure 6D illustrates the communication between all cells. Then, it shows that malignant 1 was able to send signals more effectively than malignant 2 (Fig. 6E). As illustrated, malignant1 communicate with macrophage, monocyte, B cell, T cell, epithelial cell, endothelial cell, tissue stem cell, and DC actively: it can send signal to those cells by COMPLEMENT signaling (C3-C3AR1, Fig. 6F), SPP1 signaling pathway (SPP1-CD44, Fig. 6G), ANNEXIN signaling pathway (ANXA1-FPR1, Fig. 6H), NECTIN signaling pathway (NECTIN2-TIGIT, Fig. 6I), and MIF signaling pathway (MIF-CD74 + CXCR4, Fig. 6J). Additionally, malignant1 can receive signals from those cells via MK signaling pathway (MDK-NCL, Fig. 6K), FN1 signaling pathway (FN1-CD44, Fig. 6L), LAMININ signaling pathway (LAMB3-CD44, Fig. 6M), ANGPTL signaling pathway (ANGPTL4-SDC4, Fig. 6N), and CD99 signaling pathway (CD99-CD99, Fig. 6O). Aside from MIF signaling pathway, malignant 1 influenced all other signaling pathways more than malignant 2.

In addition, all cells were divided into two groups according to their DCIRS, and the number of interactions and interaction intensity were compared between groups with high and low DCIRS cells. Low DCIRS cells communicated more often and stronger with each other than high DCIRS cells (Fig. 6P, Q). Collectively, cells with low DCIRS communicate more actively with each other compared to those with high DCIRS. This may explain why LUAD patients with low DCIRS have higher immune cell infiltration, higher immune gene expression, and higher immune pathway activity.

### Multi-omics comparison between DCIRS-high and DCIRS-low groups in TCGA-LUAD

**Fig. 7:**
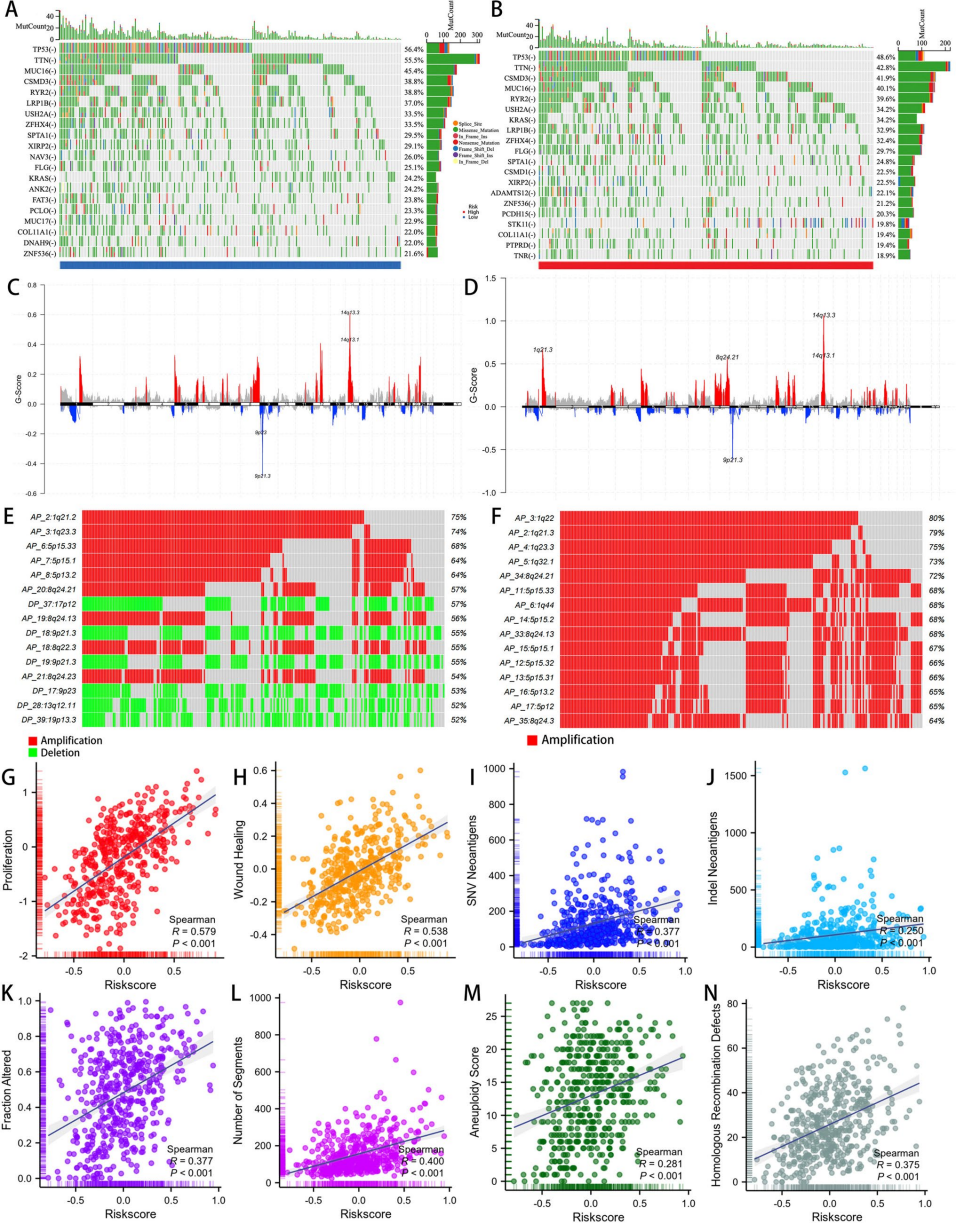
Multiomics comparisons between DCIRS high and low groups. The top 20 genes with the highest mutation frequency in the low (**A**) and high (**B**) DCIRS groups. CNV regions detected in the low (**C**) and high (**D**) DCIRS groups. The top 15 CNV events in the low (**E**) and high (**F**) DCIRS groups. DCIRS’s relationship with proliferation (**G**), wound healing (**H**), SNV neoantigens (**I**), indel neoantigens (**J**), fraction altered (**K**), number of segments (**L**), aneuploidy score (**M**), and homologous recombination defects (**N**)

We analyzed the missense mutation data and showed the top 20 genes with the highest mutation rate in the low and high DCIRS groups. It shows that TP53 and TTN mutation rates were higher in the low DCIRS group (high-DCIRS: TP53 48.6%, TTN 42.8%; low-DCIRS: TP53 56.4%, TTN 55.5%). Other genes’ mutation rates differed slightly different between two groups (Fig. 7A, B). We also compare the different CNV events between high and low DCIRS groups by GISTIC2.0 (Fig. 7C, D). The DCIRS-high group exhibited higher frequency of CNV events, and the top 15 CNV events are all amplification (Fig. 7E). In high-DCIRS group, most of the amplification occurred at chromosomal regions 1q22, 1q21.3, 1q23.3, 1q32.1 and 8q24.2. Low-DCIRS group had a significant lower frequency of CNV events, with six deletions and nine amplifications among the top 15 events (Fig. 7F). In low-DCIRS group, chromosomes 1q21.2, 1q23.3, and 5p15.33 have the most amplification, while 17p12, 9p21.3, and 9p23 have the most deletion. In addition, we also found that DCIRS and proliferation, wound healing, SNV neoantigens, Indel neoantigens, fraction altered, number of segments, aneuploidy score and homologous recombination defects were significantly positively correlated (all *p* < 0.001, Fig. 7G–N). Accordingly, patients with LUAD in the high-risk group displayed more malignant characteristics.

### DCIRS’s ability to predict ICB efficacy

**Fig. 8:**
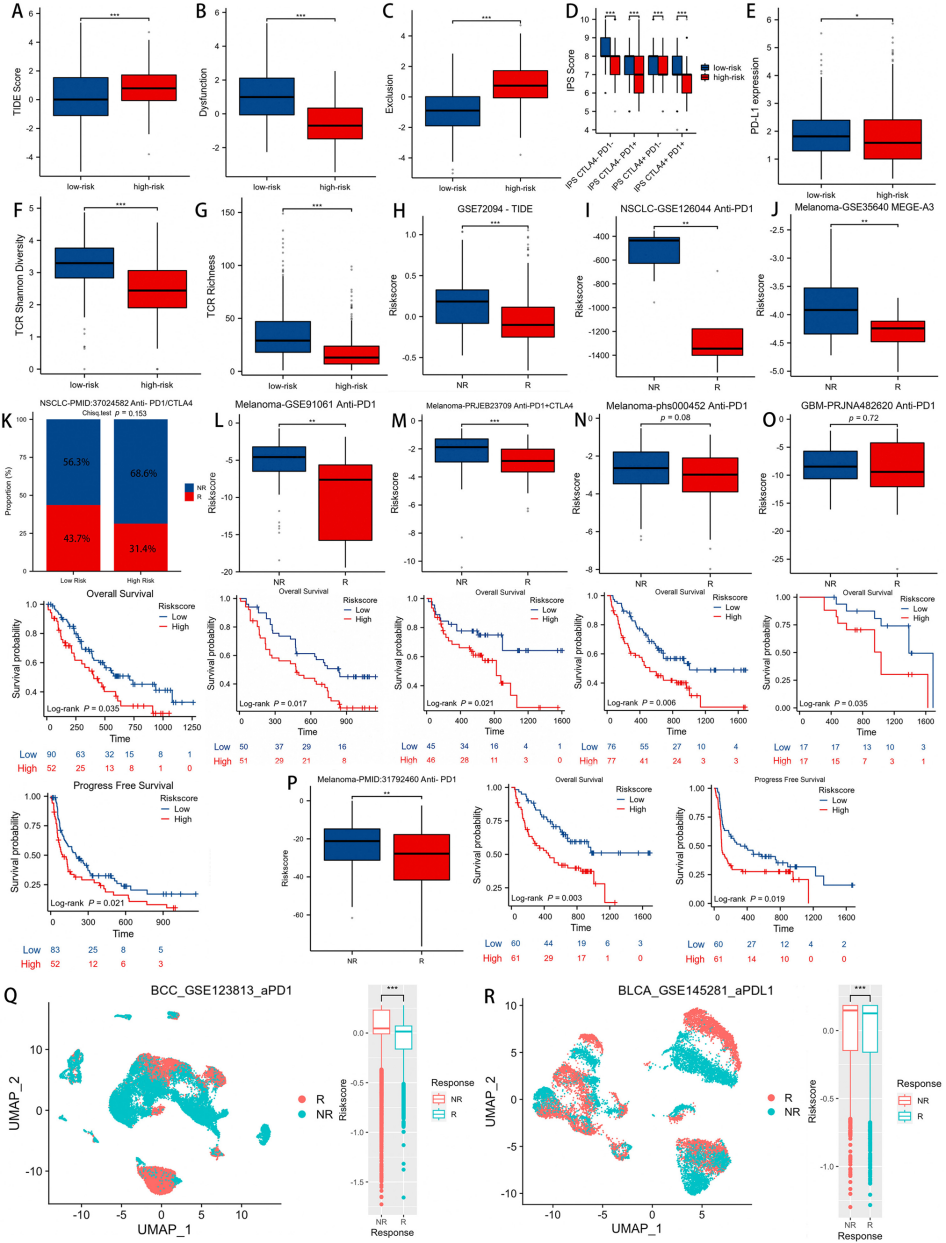
DCIRS’s ability to predict immune therapy response. Differences in TIDE (**A**), dysfunction (**B**), exclusion (**C**) and IPS (**D**) scores, PD-L1 expression (**E**), TCR Shannon diversity (**F**) and TCR richness (**G**) among high and low DCIRS groups. Differences in DCIRS between immunotherapy responders and non-responders predicted based on TIDE algorithm in the GSE72094 dataset (**H**), and GSE126044 (**I**) and GSE35640 (**J**) immunotherapy cohorts. Differences in DCIRS in cancer patients who respond to immunotherapy versus those who do not, and DCIRS’s effect on prognosis in these patients based on PMID: 37024582 (**K**), GSE91061 (**L**), PRJEB23709 (**M**), phs000452 (**N**), PRJNA482620 (**O**), and PMID: 31794260 (**P**) cohorts. UMAP reduction maps and the difference of DCIRS between Responders (R) and Non-responders (NR) to ICB therapy in GSE123813 (**Q**) and GSE145281 (**R**) datasets

Studies have shown that patients with mutations in TTN and TP53 are more susceptible to ICB therapy (Wang et al. 2021). According to our study, low-DCIRS LUAD patients had a higher frequency of TP53 and TTN mutations than high-DCIRS LUAD patients, thus it is possible that ICB therapy may have a greater effect on patients in the low-DCIRS group. A comparison of TIDE scores, exclusion scores, and dysfunction scores between DCIRS high and low groups was first conducted to verify this claim. Low-DCIRS patients showed lower TIDE scores (Fig. 8A), exclusion scores (Fig. 8C), and higher dysfunction scores (Fig. 8B). We also found that patients with low DCIRS had higher IPS scores (Fig. 8D) and higher PDL1 expression (Fig. 8E), all of which indicate that low-DCIRS patients were more sensitive to immunotherapy. It has been demonstrated that repertoire analysis of TCR can be used to stratify and monitor immunotherapy patients, and a high TCR richness is associated with a greater chance of immunotherapy being effective. TCR Shannon diversity and TCR richness were higher in patients in the low DCIRS group, which again indicated that immunotherapy was more beneficial for them (Fig. 8F, G). Compared to non-responders, DCIRS of immunotherapy responders was significantly lower in GSE72094 (Fig. 8H), GSE126044 (Fig. 8I), GSE35640 (Fig. 8J), GSE91061 (Fig. 8L), PRJEB23709 (Fig. 8M), and PMID:31792460 (Fig. 8P) cohorts. According to the PMID: 37024582, phs000452 and PRJNA482620 immunotherapy cohorts, responders had lower DCIRS than non-responders, but the difference was not significant (Fig. 8K, N, O). Additionally, immunotherapy patients with low DCIRS had a better prognosis than those with high DCIRS (Fig. 8K–P).

The findings were further confirmed by two sc-RNA sequencing datasets containing immunotherapy information. For cancer patients responding to ICB therapy, their cells had a significantly lower DCIRS than non-responders’ cells both in GSE123813 and GSE145281 datasets (Fig. 8Q, R).

In light of the above analysis, we speculate that immunotherapy may be more effective for cancer patients with low DCIRS.

### Analyses of drug sensitivity

**Fig. 9:**
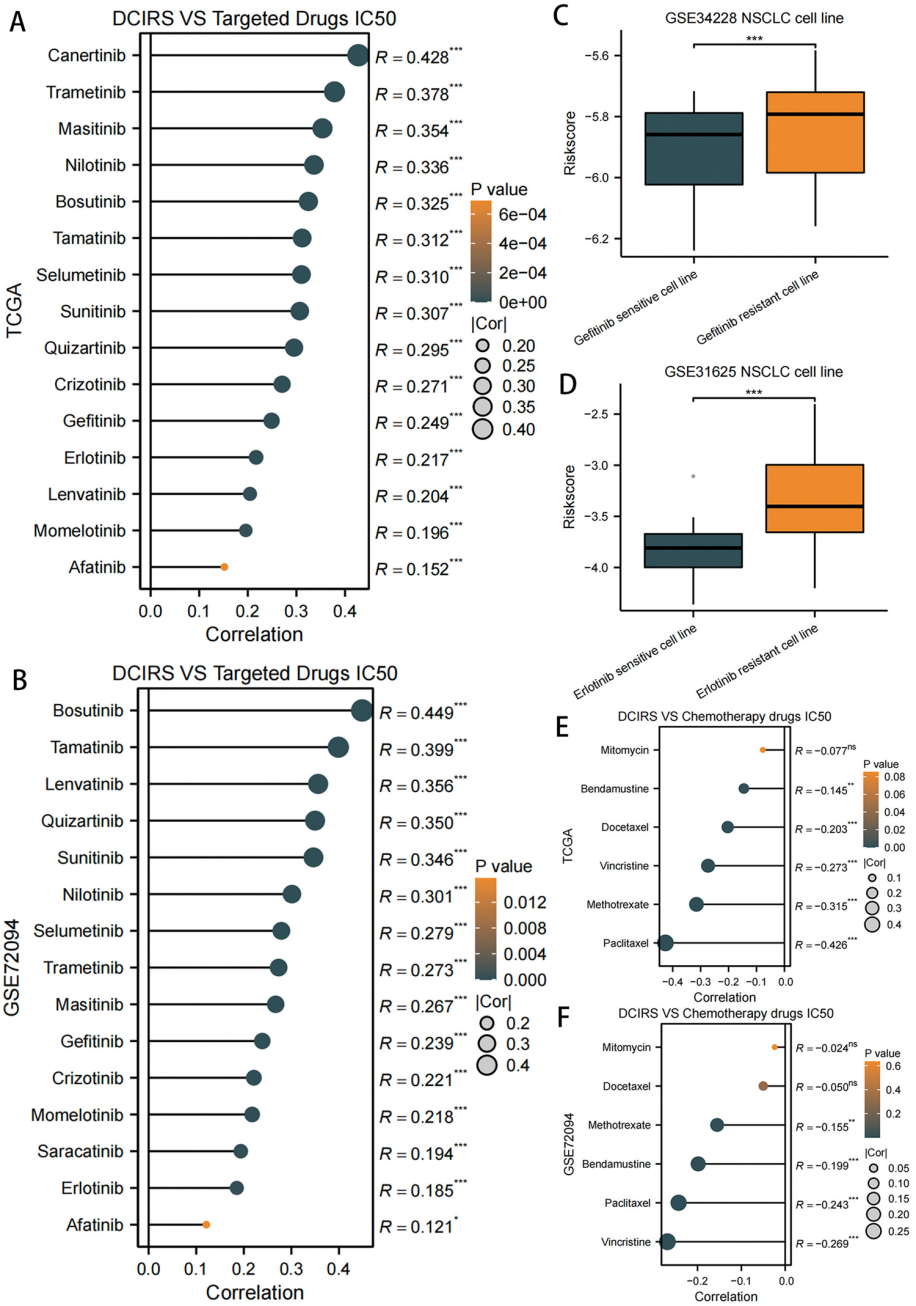
DCIRS’s relationship with targeted therapy and chemotherapy agents. DCIRS’s relationship with targeted therapy drugs’ IC50 value based on TCGA (**A**) and GSE72094 (**B**) sets. Differences of DCIRS in NSCLC cell lines sensitive to or resistant to gefitinib (**C**) or erlotinib (**D**) treatment. DCIRS’s relationship with chemotherapy drugs’ IC50 value based on TCGA (**E**) and GSE72094 (**F**) sets

On the basis of drug information in CTRP2, we examined the relationship between DCIRS and targeted therapeutic agents and chemotherapy agents. DCIRS positively correlated with the IC50 value of 15 targeted agents, including three LUAD-targeted drugs commonly used today: gefitinib, erlotinib, and crizotinib (Fig. 9A, B). This means that the lower the patient’s DCIRS, the more sensitive the patient is to targeted therapy. Besides, DCIRS were significantly lower in LUAD cell lines sensitive to gefitinib and erlotinib than in LUAD cell lines antagonistic to those two drugs (Fig. 9C, D), which further confirms our conclusion.

However, six chemotherapy drugs, including paclitaxel, docetaxel, and vincristine, commonly used to treat lung cancer, their IC50 value was significantly negatively associated with DCIRS, which means that patients with low DCIRS were resistant to chemotherapy (Fig. 9E, F).

### CTSH may be a new protective factor for LUAD

**Fig. 10:**
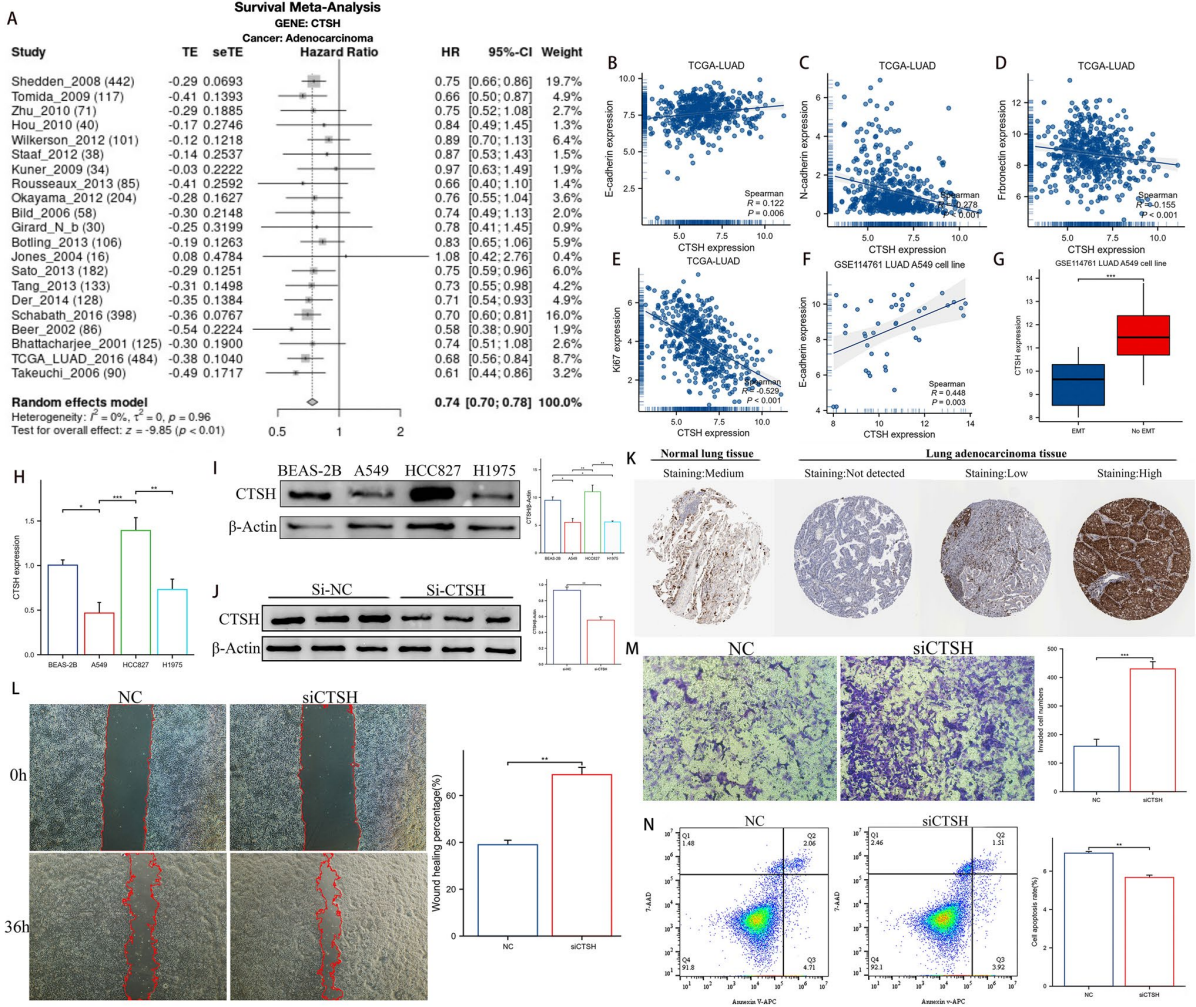
Analysis of CTSHs’ role in LUAD. **A** CTSH’s survival meta-analysis for LUAD patients. CTSH’s relationship with E-cadherin (**B**), N-cadherin (**C**), Fibronectin (**D**), and Ki67 (**E**) in TCGA-LUAD dataset. CTSH’s relationship with E-cadherin (**F**) and CTSH’s different expression pattern between EMT A549 cells and noEMT A549 cells (**G**) in GSE114761 dataset. qRT-PCR (**H**) and Western blotting (**I**) to explore CTSH’s level between different cell lines. **J** si-RNA’s transfection efficacy. **K** Representative immunohistochemistry staining for CTSH in normal lung tissues and LUAD tissues. The difference of HCC827 cells’ migration (**L**), invasion (**M**) ability, and apoptosis rates (**N**) between NC and si-CTSH groups

We found that only CTSH's function in LUAD is unclear out of the seven genes that make up DCIRS, so we decided to explore its role in LUAD further. According to survival meta-analysis, CTSH is protective against LUAD in most studies (HR < 1, Fig. 10A). In addition, we examined the relationship between CTSH and EMT-related markers in the TCGA database, such as E-cadherin (Fig. 10B), N-cadherin (Fig. 10C), and fibronectin (Fig. 10D). CTSH was positively correlated with E-cadherin, while negatively correlated with N-cadherin and fibronectin. As cells undergo EMT, the content of E-cadherin decreases, while the content of N-cadherin and Fibronectin increases. The relationship between CTSH and these markers suggests that it may inhibit cells’ EMT, and the data in GSE114651 set containing LUAD A549 cells’ EMT data confirmed it (Fig. 10F, G). Additionally, CTSH correlated negatively with Ki67, a proliferation marker for cancer cells (Fig. 10E). Based on the above results, CTSH might inhibit the proliferation and EMT phenotypes of LUAD (Pearson 2019; Mrozik et al. 2018; Li et al. 2017; Sun and Kaufman 2018).

As a next step, we analyzed the expression pattern of CTSH in cells. qRT-PCR showed that CTSH was expressed at its highest level in HCC827 cells, followed by BEAS-2B, and at the lowest level in A549s and H1975 cells (Fig. 10H). Western blotting generated similar results with qRT-PCR (Fig. 10I). To examine CTSH expression at tissue levels, we acquired immunohistochemical data from the HPA database. We found that as CTSH is mainly expressed in the cytoplasm of cells, normal lung tissue showed moderate CTSH staining, while LUAD tissue showed undetected, low, or high CTSH staining (Fig. 10K).

Considering the expression level of CTSH, HCC827 cells were chosen for further study. In HCC827 cells, the expression of CTSH was significantly reduced by siRNA’s knockdown (Fig. 10J). The knockdown of CTSH improved the migration (Fig. 10L) and invasion (Fig. 10M) abilities of HCC827 cells, but inhibited its apoptosis (Fig. 10 N).

As a result, CTSH may serve as a new protective factor in LUAD.

## Discussion

A significant stride forward in research into tumor-infiltrating immunocytes in the TME has been made possible due to advances in scRNA-seq technology. A majority of current studies focus on adaptive immunocytes, while the role of innate immunocytes is understudied, which may have a marked impact on prognosis and treatment response for cancer patients. A vital component of innate immunity are dendritic cells (DCs), which play a significant role in the development of many types of malignant. It has been found that DC infiltration is important for the sensitivity of immunotherapy, and thus DC-dependent vaccines have been developed for immunotherapy in recent years. There is, however, a lack of comprehensive research on DC’s role in LUAD development. Through the use of sc-RNA sequencing technology, many studies have developed gene signatures based on marker genes of immunocytes, such as B and NK cells (Song et al. 2022a, b). In light of these studies, a novel dendritic cell implicated risk score (DCIRS) based on DC marker genes was developed for LUAD patients. DCIRS-high patients have a poorer prognosis, less immune cell infiltration, and less sensitivity to immunotherapy than patients with low DCIRS.

DCIRS is composed of seven DC marker genes (IL7R, IRF8, CLEC10A, CLEC2D, CTSH, ALDH2, and NDRG2), most of which are associated with prognosis in patients with LUAD. Compared with normal lung tissues, LUAD tissues express lower levels of IL7R, which results in a worse prognosis. Additionally, IL7R was significantly associated with immune infiltration in LUAD (Wang et al. 2022). It has also been observed that the expression of IRF8 is reduced in LUAD due to abnormal hypermethylation and that IRF8 expression is associated with immune infiltration (Suzuki et al. 2014; Long et al. 2021). IRF8 is also a tumor suppressor, and it has the ability to induce senescence in lung cancer cells (Liang et al. 2019). Currently, CLEC10A is being investigated as a cancer immunotherapy target due to its ability to modulate innate and adaptive immunity. CLEC10A also has diagnostic, prognostic, and immune-implicated significance in lung adenocarcinoma (He et al. 2021; Qin et al. 2022). Research has previously linked LUAD with ALDH2, a member of the superfamily of aldehyde dehydrogenase. When ALDH2 was overexpressed, LUAD cells showed decreased stemness, migration, and proliferation, whereas when ALDH2 was knocked down, these characteristics were increased. As ALDH2 being repressed, acetaldehyde (ACE) accumulated, increasing migration features of LUAD cells and causing DNA damage (Li et al. 2019a). NDRG2 is a tumor suppress gene that inhibits cancer progression. Transwell assays revealed that A549 cells overexpressed NDRG2 had a decreased migration and invasion rates (Faraji et al. 2015). The above 5 genes all demonstrated tumor inhibition function, demonstrating DC’s role in anti-tumor immunity. As well, an increased expression of CLEC2D led to better prognoses in multiple cohorts of patients with LUAD, suggesting that this protein may act as a protective mechanism (Braud et al. 2018). The role of CTSH in LUAD, however, has not been extensively studied, and its value in LUAD remains unclear, so we decided to unravel its role in LUAD. We found CTSH may inhibit cells’ proliferation and EMT using bioinformatics, and in vitro experiments showed that after CTSH was knockdown, HCC827 cells’ migration and invasion ability increased, while apoptosis decreased. So CTSH may exerts its protective effect by inhibiting LUAD cells’ proliferation, EMT, migration, and invasion, while promoting LUAD cells’ apoptosis. The role of CTSH in LUAD and its mechanism have never been explored before. For the first time, we demonstrate its role and provide a reference for future research.

Across one training and six validation sets, DCIRS showed high predictive power for the prognosis of LAUD patients. We investigated the potential mechanisms behind DCIRS due to its satisfactory predictive capability. First, we found that patients with high DCIRS expressed higher levels of TOP2A and FAM83A. FAM83A and TOP2A overexpression lead to significantly worse prognoses for patients with LUAD, and knockdown of TOP2A of FAM83A significantly reduce LUAD cell’s malignant phenotype (Zhang et al. 2019; Yu et al. 2020; Du et al. 2020; Kou et al. 2020). Additionally, GO and GSEA analysis indicated that the gene sets enriched in the high DCIRS group are primarily involved in chromosome division and cell cycle pathways, which plays a crucial role in the proliferation and aggression of cancers (Ford and Pardee 1999). In low DCIRS group, protective factors such as SFTPC (Li et al. 2019b) and SUSD2 (Guo et al. 2020) were highly expressed, and gene sets enriched in the low DCIRS group mainly involved in immune-implicated functions, such as adaptive immunity and antigen presentation. Additionally, GSVA analysis showed that low DCIRS groups displayed higher activity in multiple immune-related pathways. In addition, we compared the differences in immune cell infiltration levels between high and low DCIRS groups using six different quantification methods, including ssGSEA, EPIC, MCPcounter, estimate, Quantiseq, and TIMER. Patients with low DCIRS showed higher immune cell infiltration, similar to results of GSVA enrichment. When immune cells are infiltrated at a low level, tumor cells can escape immune surveillance, facilitating the progression of the tumor. There might be a connection between this and the significantly reduced survival of LUAD patients with high-DCIRS.

Cell–cell communication analysis was conducted to determine the mechanism behind the disparity in immune infiltration between the low and high DCIRS groups. First, we calculated the DCIRS for each cell and found that malignant cells posed the greatest risk, and Malignant1 had a significantly lower risk score than Malignant2. Following that, we predicted several possible interactions between LUAD cells and immune cells. We found that Malignant1 can communicate with cells in TME through COMPLEMENT, SPP1, ANNEXIN, NECTIN, MIF, MK, FN1, LAMININ, ANGPTL, and CD99 signaling pathways, and in all pathways except MIF, Malignant1 played a greater role than Malignant2. Many biological functions are regulated by these signaling pathways, including metabolism, pyroptosis, antitumor immunity, and immune checkpoint expression (Zhang et al. 2020; Vacchelli et al. 2015; Baracco et al. 2019; Pio et al. 2019; Kwak et al. 2018; Shin et al. 2020; Ives et al. 2021; Gasparrini and Audrito 2022; Ferrarelli 2022). We also divided all cells into low and high DCIRS groups based on their DCIRS and found that cells in the low DCIRS group had a higher number of interactions and greater interaction strength between each other than the cells in the high DCIRS group. Due to this, we speculate that LUAD patients with low DCIRS have higher immune activity than those with high DCIRS, possibly because their cells communicate with TME more actively.

As a result of the disparity in immune cell infiltration and gene mutations between different risk groups, we explored the value of the DCIRS in predicting immune therapy response. First, by using TIDE and IPS predictive algorithms and comparing PD-L1 expression and TCR richness between the two groups, we found that patients with lower DCIRS may benefit more from ICB therapy. To further confirm this, we collected eight independent immunotherapy cohorts and found that immunotherapy responders had lower DCIRS than non-responders in each of these cohorts, although in PMID: 37024582, phs000452 and PRJNA482620 cohorts the difference was insignificant. Additionally, patients with low DCIRS had significantly better prognosis than those with high DCIRS. It appears that immunotherapy is more likely to benefit patients with a low DCIRS, and it may be possible to use DCIRS to predict immunotherapy response if further validation is obtained.

Finally, the correlation between DCIRS and drugs’ IC50 value was examined. Based on our findings, DCIRS and IC50 value of multiple targeted therapy agents showed a significant positive correlation, suggesting that patients with low DCIRS might more likely to benefit from targeted therapy. Moreover, NSCLC cell lines sensitive to gefitinib and erlotinib, which are two commonly used targeted therapy drugs for treating NSCLC, had significantly lower DCIRS than those resistant to them. There is, however, a significant negative correlation between DCIRS and chemotherapy drugs’ IC50 value, which may mean patients with low DCIRS are less sensitive to chemotherapy.

However, several limitations reduce the validity of this study. First, this DCIRS was constructed using public datasets. A large-scale prospective clinical trial will be necessary to confirm the predictive value. Another limitation of this study is the absence of in vivo experiments that would have contributed to a deeper understanding of CTSH’s molecular mechanisms.

Collectively, dendritic cell implicated risk score (DCIRS) proved to be a powerful prognostic and immune therapy predictor for LUAD patients. It may aid in the development of personalized treatments in the future.

### Supplementary Information

Below is the link to the electronic supplementary material.

Supplementary file1 (DOCX 46 KB)

## Data Availability

The datasets presented in this study can be found in online repositories. The names of the repository/repositories and accession number(s) can be found in the article/supplementary material.
