# Supplementary material for: Machine-learning and combined analysis of single-cell and bulk-RNA sequencing identified a DC gene signature to predict prognosis and immunotherapy response for patients with lung adenocarcinoma
Source: J Cancer Res Clin Oncol. 2023 Jul 28;149(15):13553–74. doi: 10.1007/s00432-023-05151-w (PMC10590321; doi:10.1007/s00432-023-05151-w)
Supplement: Supplementary file 1 — Supplementary file1 (DOCX 46 KB) [file 432_2023_5151_MOESM1_ESM.docx]

**Table S1. 7 datasets used for analysis in our study.**

| **Characteristics** | **TCGA** | **GSE72094** | **GSE31210** | **GSE11969** | **GSE41271** | **GSE42127** | **GSE50081** |
| --- | --- | --- | --- | --- | --- | --- | --- |
| **Sample Size(n)** | 497 | 397 | 226 | 90 | 183 | 133 | 127 |
| **Age(years)** |  |  |  |  |  |  |  |
| ≤65 | 236 | 118 | 176 | 59 | - | 63 | 40 |
| >65 | 251 | 279 | 50 | 31 | - | 70 | 87 |
| **Gender** |  |  |  |  |  |  |  |
| female | 269 | 221 | 121 | 43 | 93 | 68 | 65 |
| male | 228 | 176 | 105 | 47 | 90 | 65 | 62 |
| **TNM Stage** |  |  |  |  |  |  |  |
| Stage I | 267 | 253 | 168 | 52 | 101 | 89 | 92 |
| Stage II | 118 | 67 | 58 | 13 | 28 | 22 | 35 |
| Stage III | 80 | 57 | 0 | 25 | 49 | 21 | 0 |
| Stage IV | 25 | 15 | 0 | 0 | 5 | 0 | 0 |
| NA | 7 | 5 | 0 | 0 | 0 | 1 | 0 |
| **OS event** |  |  |  |  |  |  |  |
| dead | 180 | 112 | 35 | 40 | 71 | 43 | 51 |
| alive | 317 | 285 | 191 | 50 | 112 | 90 | 76 |

**Table S2.** **DC** **cell** **marker** **genes** **identified** **from** **EMTAB6149.**

| No. | Gene | No. | Gene | No. | Gene |
| --- | --- | --- | --- | --- | --- |
| 1 | PLD4 | 32 | AIF1 | 63 | PPA1 |
| 2 | S100B | 33 | IGSF6 | 64 | SLA |
| 3 | CD1C | 34 | C1orf54 | 65 | IL7R |
| 4 | PPP1R14A | 35 | KCNMB1 | 66 | GIMAP4 |
| 5 | PKIB | 36 | IL18 | 67 | EVL |
| 6 | CSF2RA | 37 | CLEC4A | 68 | PPP2R5C |
| 7 | FCER1A | 38 | GAPT | 69 | NKG7 |
| 8 | CD1A | 39 | HLA-DRB5 | 70 | CLEC2D |
| 9 | SERPINF1 | 40 | C12orf45 | 71 | GIMAP7 |
| 10 | RNASE6 | 41 | FGL2 | 72 | CD69 |
| 11 | IDO1 | 42 | CST3 | 73 | CTSW |
| 12 | CPVL | 43 | CXorf21 | 74 | PIK3IP1 |
| 13 | CD1E | 44 | CLEC10A | 75 | CD2 |
| 14 | HLA-DQB2 | 45 | CD86 | 76 | ITM2A |
| 15 | PLAC8 | 46 | LY86 | 77 | LCK |
| 16 | LGALS2 | 47 | CD83 | 78 | CD3E |
| 17 | IRF8 | 48 | TYROBP | 79 | CD7 |
| 18 | HCK | 49 | HLA-DMA | 80 | TRBC2 |
| 19 | PAK1 | 50 | DAPP1 | 81 | IL32 |
| 20 | LST1 | 51 | ALDH2 | 82 | GZMA |
| 21 | GSN | 52 | RAB32 | 83 | CD3D |
| 22 | SPI1 | 53 | IFI30 |  |  |
| 23 | MS4A6A | 54 | CLIC2 |  |  |
| 24 | NDRG2 | 55 | CFP |  |  |
| 25 | HLA-DQA1 | 56 | MNDA |  |  |
| 26 | CALHM6 | 57 | LGALS9 |  |  |
| 27 | HLA-DQA2 | 58 | CTSH |  |  |
| 28 | HLA-DMB | 59 | CLN8 |  |  |
| 29 | SMCO4 | 60 | TSPAN13 |  |  |
| 30 | PLEK | 61 | GRN |  |  |
| 31 | HLA-DQB1 | 62 | CD300A |  |  |

**Table S3. Overall survival associated DC marker genes in LUAD patients from TCGA.**

| No. | Gene | HR | *p* value | No. | Gene | HR | *p* value |
| --- | --- | --- | --- | --- | --- | --- | --- |
| 1 | GIMAP7 | 0.582 | 0.001 | 18 | NDRG2 | 0.661 | 0.007 |
| 2 | CLEC10A | 0.586 | 0.001 | 19 | HLA-DRB5 | 0.667 | 0.007 |
| 3 | CFP | 0.594 | 0.001 | 20 | LST1 | 0.667 | 0.007 |
| 4 | HLA-DMA | 0.602 | 0.001 | 21 | PIK3IP1 | 0.668 | 0.007 |
| 5 | IRF8 | 0.668 | 0.008 | 22 | IL7R | 0.668 | 0.008 |
| 6 | HLA-DMB | 0.612 | 0.001 | 23 | HLA-DQA1 | 0.676 | 0.009 |
| 7 | GAPT | 0.616 | 0.001 | 24 | PLEK | 0.684 | 0.011 |
| 8 | PLD4 | 0.617 | 0.001 | 25 | FCER1A | 0.703 | 0.019 |
| 9 | GIMAP4 | 0.622 | 0.002 | 26 | MNDA | 0.709 | 0.020 |
| 10 | CLEC2D | 0.682 | 0.011 | 27 | CD300A | 0.703 | 0.020 |
| 11 | FGL2 | 0.624 | 0.002 | 28 | AIF1 | 0.712 | 0.024 |
| 12 | CD2 | 0.633 | 0.002 | 29 | EVL | 0.718 | 0.028 |
| 13 | IGSF6 | 0.650 | 0.004 | 30 | CD69 | 0.725 | 0.032 |
| 14 | CTSH | 0.660 | 0.005 | 31 | CPVL | 0.729 | 0.035 |
| 15 | CD1E | 0.654 | 0.005 | 32 | HCK | 0.731 | 0.036 |
| 16 | LY86 | 0.663 | 0.006 | 33 | MS4A6A | 0.724 | 0.039 |
| 17 | ALDH2 | 0.660 | 0.006 |  |  |  |  |

**Table S4. Univariable and multivariable Cox regression analysis of the DCIRS in TCGA cohort.**

| **Characteristics** | **Univariate analysis** | |  | **Multivariate analysis** | |
| --- | --- | --- | --- | --- | --- |
|  | **Hazard ratio (95% CI)** | **P value** |  | **Hazard ratio (95% CI)** | **P value** |
| **Age (**≤65 vs >65) | 1.220 (0.907 - 1.642) | 0.188 |  |  |  |
| **Gender** (male vs female) | 1.047 (0.781 - 1.404) | 0.758 |  |  |  |
| **TNM Stage** (I&II vs III&IV) | 2.631 (1.926 - 3.595) | **< 0.001** |  | 2.324 (1.690 - 3.196) | **< 0.001** |
| **DCIRS**(low vs high) | 1.942 (1.438 - 2.624) | **< 0.001** |  | 1.713 (1.257 - 2.335) | **< 0.001** |
|  |  |  |  |  |  |

**Table S5. Univariable and multivariable Cox regression analysis of the DCIRS in GSE72094 cohort.**

| **Characteristics** | **Univariate analysis** | |  | **Multivariate analysis** | |
| --- | --- | --- | --- | --- | --- |
|  | **Hazard ratio (95% CI)** | **P value** |  | **Hazard ratio (95% CI)** | **P value** |
| **Age (**≤65 vs >65) | 0.895 (0.589 - 1.360) | 0.604 |  |  |  |
| **Gender** (male vs female) | 1.139 (0.779 - 1.666) | 0.501 |  |  |  |
| **TNM Stage** (I&II vs III&IV) | 1.390 (0.882 - 2.189) | 0.156 |  |  |  |
| **DCIRS** (low vs high) | 2.715 (1.800 - 4.096) | **< 0.001** |  | 2.715 (1.800 - 4.096) | **< 0.001** |
|  |  |  |  |  |  |

**Table S6. Univariable and multivariable Cox regression analysis of the DCIRS in GSE11969 cohort.**

| Characteristics | **Univariate analysis** | |  | **Multivariate analysis** | |
| --- | --- | --- | --- | --- | --- |
|  | **Hazard ratio (95% CI)** | **P value** |  | **Hazard ratio (95% CI)** | **P value** |
| **Age (**≤65 vs >65) | 1.606 (0.857 - 3.011) | 0.139 |  |  |  |
| **Gender** (male vs female) | 1.332 (0.714 - 2.486) | 0.367 |  |  |  |
| **TNM Stage** (I&II vs III) | 3.007 (1.608 - 5.622) | **< 0.001** |  | 3.246 (1.730 - 6.087) | **< 0.001** |
| **DCIRS**(low vs high) | 2.186 (1.151 - 4.154) | **0.017** |  | 2.389 (1.254 - 4.552) | **0.008** |
|  |  |  |  |  |  |

**Table S7. Univariable and multivariable Cox regression analysis of the DCIRS in GSE50081 cohort.**

| Characteristics | **Univariate analysis** | |  | **Multivariate analysis** | |
| --- | --- | --- | --- | --- | --- |
|  | **Hazard ratio (95% CI)** | **P value** |  | **Hazard ratio (95% CI)** | **P value** |
| **Age (**≤65 vs >65) | 0.687 (0.366 - 1.292) | 0.244 |  |  |  |
| **Gender** (male vs female) | 0.709 (0.406 - 1.240) | 0.228 |  |  |  |
| **TNM Stage** (I vs II) | 2.443 (1.383 - 4.316) | **0.002** |  | 2.268 (1.278 - 4.022) | **0.005** |
| **DCIRS** (low vs high) | 2.164 (1.217 - 3.847) | **0.009** |  | 2.012 (1.127 - 3.590) | **0.018** |
|  |  |  |  |  |  |

**Table S8. Univariable and multivariable Cox regression analysis of the DCIRS in GSE41271 cohort.**

| **Characteristics** | **Univariate analysis** | |  | **Multivariate analysis** | |
| --- | --- | --- | --- | --- | --- |
|  | **Hazard ratio (95% CI)** | **P value** |  | **Hazard ratio (95% CI)** | **P value** |
| **Gender** (male vs female) | 0.592 (0.366 - 0.957) | 0.032 |  | 0.682 (0.419 - 1.110) | 0.124 |
| **TNM Stage** (I&II vs III&IV) | 2.653 (1.650 - 4.267) | **< 0.001** |  | 2.585 (1.608 - 4.155) | **< 0.001** |
| **DCIRS** (low vs high) | 1.800 (1.117 - 2.900) | **0.016** |  | 1.675 (1.033 - 2.716) | **0.036** |
|  |  |  |  |  |  |

**Table S9. Univariable and multivariable Cox regression analysis of the DCIRS in GSE42127 cohort.**

| **Characteristics** | **Univariate analysis** | |  | **Multivariate analysis** | |
| --- | --- | --- | --- | --- | --- |
|  | **Hazard ratio (95% CI)** | **P value** |  | **Hazard ratio (95% CI)** | **P value** |
| **Age (**≤65 vs >65) | 0.697 (0.376 - 1.291) | 0.251 |  |  |  |
| **Gender** (male vs female) | 0.554 (0.295 - 1.043) | 0.067 |  | 0.660 (0.347 - 1.254) | 0.205 |
| **TNM Stage** (I&II vs III) |  | 0.123 |  |  |  |
| **DCIRS** (low vs high) | 2.927 (1.518 - 5.643) | **0.001** |  | 2.714 (1.396 - 5.279) | **0.003** |
|  |  |  |  |  |  |

**Table S10. Univariable and multivariable Cox regression analysis of the DCIRS in GSE31210cohort.**

| **Characteristics** | **Univariate analysis** | |  | **Multivariate analysis** | |
| --- | --- | --- | --- | --- | --- |
|  | **Hazard ratio (95% CI)** | **P value** |  | **Hazard ratio (95% CI)** | **P value** |
| **Age (**≤65 vs >65) | 2.583 (1.313 - 5.083) | **0.006** |  | 3.460 (1.714 - 6.981) | **< 0.001** |
| **Gender** (male vs female) |  | 0.218 |  |  |  |
| **TNM Stage** (Ivs II) | 3.976 (2.044 - 7.733) | **< 0.001** |  | 4.361 (2.205 - 8.625) | **< 0.001** |
| **DCIRS** (low vs high) | 2.114 (1.051 - 4.250) | **0.036** |  | 2.078 (1.021 - 4.229) | **0.044** |
|  |  |  |  |  |  |
